# Higher methylation subtype of malignant melanoma and its correlation with thicker progression and worse prognosis

**DOI:** 10.1002/cam4.3127

**Published:** 2020-05-14

**Authors:** Yosuke Yamamoto, Keisuke Matsusaka, Masaki Fukuyo, Bahityar Rahmutulla, Hiroyuki Matsue, Atsushi Kaneda

**Affiliations:** ^1^ Department of Dermatology Graduate School of Medicine Chiba University Chiba Japan; ^2^ Department of Molecular Oncology Graduate School of Medicine Chiba University Chiba Japan; ^3^ Department of Genome Research and Development Kazusa DNA Research Institute Chiba Japan

**Keywords:** DNA methylation, epigenotype, invasion, malignant melanoma, skin neoplasm

## Abstract

Malignant melanoma (MM) is the most life‐threatening disease among all skin malignancies, and recent genome‐wide studies reported *BRAF*, *RAS*, and *NF1* as the most frequently mutated driver genes. While epigenetic aberrations are known to contribute to the oncogenic activity seen in various cancers, their role in MM has not been fully investigated. To investigate the role of epigenetic aberrations in MM, we performed genome‐wide DNA methylation analysis of 51 clinical MM samples using Infinium 450k beadarray. Hierarchical clustering analysis stratified MM into two DNA methylation epigenotypes: high‐ and low‐methylation subgroups. Tumor thickness was significantly greater in case of high‐methylation tumors than low‐methylation tumors (8.3 ± 5.3 mm vs 4.5 ± 2.9 mm, *P* = .003). Moreover, prognosis was significantly worse in high‐methylation cases (*P* = .03). Twenty‐seven genes were found to undergo significant and frequent hypermethylation in high‐methylation subgroup, where *TFPI2* was identified as the most frequently hypermethylated gene. MM cases with lower expression levels of *TFPI2* showed significantly worse prognosis (*P* = .001). Knockdown of *TFPI2* in two MM cell lines, CHL‐1 and G361, resulted in significant increases of cell proliferation and invasion. These indicate that MM can be stratified into at least two different epigenetic subgroups, that the MM subgroup with higher DNA methylation shows a more progressive phenotype, and that methylation of *TFPI2* may contribute to the tumor progression of MM.

## Graphical abstract

We conducted DNA methylome analysis using 51 clinical malignant melanoma samples, and focused on aberrant DNA hypermethylation in promoter CpG islands, which are critical regions that result in gene silencing. We found that malignant melanoma can be stratified into at least two subgroups based on promoter methylation status, and that High‐methylation subgroup involving *TFPI2* methylation exhibits advanced tumor phenotypes, characterized by thicker progression and worse prognosis.

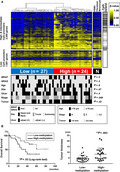

**Abbreviations**
CSD: chronically sun‐damaged skin
Infinium 450k: Infinium HumanMethylation450 BeadChip
MM: malignant melanoma
PRC: polycomb repressive complex
TCGA: The Cancer Genome Atlas
TSS: transcriptional start site
UV: ultraviolet

## 1 INTRODUCTION

Malignant melanoma (MM) is a skin neoplasm that was relatively rare a century ago; however, globally, the prevalence of the cancer has been increasing over the last 40 years.^1^, ^2^ While MM is curable by surgery at an early stage, advanced forms of the disease are considered fatal and difficult to treat. Cost of diagnosis and treatment of MM are 10 times greater than other skin cancers.^3^ Moreover, the incidence rates of MM are estimated to increase by 3%‐7% per year, and are expected to double in 10‐20 years.^4^ The incidence rate per 100 000 people per year varies widely from country to country: 35‐40 among Australians, 15‐20 among Americans,^5^ and <5 among Japanese individuals. The occurrence sites are also different such that Asians often present with acral lesions.^6^ These differences are thought to be due to genetic factors as well as environmental factors, such as exposure to sunlight, that make significant contributions to the tumorigenesis that occurs.

Tumors arise through accumulation of genomic and epigenomic aberrations.^7^, ^8^, ^9^ As for genomic aberrations, 66% of MM reportedly exhibited somatic *BRAF* mutations, of which 80% had V600E mutation, and mutated *BRAF* was shown to be expressed at higher levels.^10^ *KIT* mutations were found frequently in acral cases at 36%, but not in MM on chronic sun‐damaged (CSD) areas.^11^ Comprehensive genomic analysis on MM was recently conducted, including that of TCGA, and revealed driver gene mutations of MM, such as *BRAF*, *NRAS*, and *NF1*.^12^, ^13^, ^14^

As for epigenomic aberrations, DNA hypermethylation of promoter CpG islands suppresses the expression of downstream genes, and such methylation‐associated gene silencing is known to be one of the major mechanisms to inactivate tumor suppressor genes in cancer.^15^, ^16^, ^17^ As for tumor suppressor genes, *PTEN* and *p16* were reportedly hypermethylated in MM.^18^, ^19^ Methylation of several marker genes was reported in a genome‐wide methylation study that showed significant association between methylation phenotype and prognosis.^20^ *COL1A2* and *DDIT4L* were significantly methylated in advanced MM compared with nevi and early MM samples. Thomas *et al* reported that 47 cases of primary MM were stratified into three groups by methylation pattern, and the most highly methylated group was significantly associated with thicker tumors.^21^ Lauss *et al* stratified 50 metastatic MMs into three groups based on methylation patterns, and also reported a correlation with prognosis.^22^ The most highly methylated group was associated with a significantly worse prognosis.

The Cancer Genome Atlas (TCGA) conducted large‐scale comprehensive analysis of MM,^12^ which stratified MM into *BRAF*‐mutated, *RAS*‐mutated, *NF1*‐mutated, and triple wild‐type subtypes. For DNA methylation, they conducted Infinium assays and classified MM into four distinct DNA methylation epigenotypes using all the CpG sites in the entire genome. There was a significant difference in prognosis between the most highly methylated subtype and normal‐like subtype. Although the data suggested that aberrant DNA methylation might contribute to tumor prognosis of MM, association of DNA methylation in promoter CpG islands and its tumorigenic contribution in MM are yet to be fully clarified.

Therefore, in this present study, we conducted DNA methylome analysis using 51 clinical MM samples, and focused on aberrant DNA hypermethylation in promoter CpG islands, which are critical regions that result in gene silencing. We found that MM is stratified into at least two molecular subtypes based on promoter hypermethylation: high‐ and low‐methylation subgroups. High‐methylation subgroup significantly correlates with more advanced tumors with greater tumor thickness and worse prognosis, and silencing of one of the most frequently methylated genes, *TFPI2*, is found to increase cell proliferation and invasion.

## 2 MATERIALS AND METHODS

### 2.1 Clinical samples and cell lines

Clinical specimens were obtained from 77 patients with MM who underwent therapy at Chiba University Hospital from 2011 to 2016; written informed consent was obtained. The surgical specimens were preserved as formalin‐fixed paraffin‐embedded (FFPE) samples. Specimens were diagnosed microscopically by two independent pathologists. All samples were dissected to have >50% of tumor cell content. Melanoma cell lines, CHL‐1 and G361, and primary human melanocytes were obtained from the American Type Culture Collection and freshly used for this study. Mycoplasma contamination was tested using CycleavePCR Mycoplasma Detection Kit (Takara Bio) to confirm mycoplasma‐free. DNA was extracted using the QIAamp DNA FFPE Tissue Kit (Qiagen). The study protocol was approved by the institutional review board at Chiba University.

### 2.2 Hotspot mutation analysis of BRAF and NRAS

For the 77 MM samples, hotspot mutations of *BRAF* (codon 600) and *NRAS* (codons 12, 13, and 61) were analyzed using pyrosequencing assay through the PyroMark Q 96 (Qiagen) kit, following the manufacturer's protocol. Primers were designed as previously reported^23^ (Table S1). From each sample, 2 ng of DNA was amplified by PCR, and 20 μL of each PCR product was used for the subsequent pyrosequencing analysis.

### 2.3 Infinium assays

Infinium HumanMethylation450 BeadChip (Infinium 450k) (Illumina), a comprehensive methylation analysis technique, analyzes more than 450 000 methylated CpG sites covering 99% of Refseq genes. Of the 77 samples, sufficient DNA was obtained from 56 samples, which underwent DNA quality checks using the FFPE QC Kit (Illumina). Fifty‐one samples exhibited satisfactory quality and were used for the subsequent analyses. After FFPE DNA Restoration assay was performed on 250 ng of DNA extracted from FFPE specimens, bisulfite conversion was performed using the Zymo EZ DNA Methylation Kit (Zymo Research), and the bisulfite‐treated DNA was subsequently used for DNA methylation analysis. Infinium analysis was performed on 51 clinical specimens as well as normal melanocyte. As methylation analysis focused on the promoter region, the probe nearest to the transcription start site (TSS) in the gene was regarded as a representative probe of the gene.^24^ The CpG score of each probe was calculated as previously reported.^25^ Control samples of 0%, 25%, 50%, 75%, and 100% methylation were prepared as previously reported,^26^ and also underwent Infinium analysis to exclude probes without high accuracy in quantitative methylation measurement. Infinium 450k data of human peripheral blood mononuclear cells and human normal fibroblast were previously obtained.^27^

### 2.4 Short hairpin RNA (shRNA) knockdown

shRNAs against *TFPI2* (shTFPI2_#1 and _#2) were constructed using lentiviral vectors encoding TFPI2‐specific shRNA to downregulate and eliminate its expression. Control shRNA (shNON) was used as a nontarget control. Vector construction was performed as previously reported^28^ using pLKO.1‐puro vector (Sigma‐Aldrich), and inserted oligonucleotide sequences are listed in Table S2. The shRNA lentiviral vector was packaged with the appending vectors, psPAX2 and pMD2, in HEK 293 T cells using FuGENE 6 (Roche, Mannheim, Germany). CHL‐1 and G361 cell lines were cultured in Dulbecco's Modified Eagle Medium (DMEM), supplemented with 10% fetal bovine serum (FBS) and 1% Penicillin‐Streptomycin. Cells were infected with the generated lentiviruses and then selected by exposure to 1.0 g/mL of puromycin (Sigma‐Aldrich) for 3 days.

### 2.5 Reverse‐transcription quantitative PCR (RT‐qPCR)

cDNA was synthesized by reverse transcription of 500 ng of total RNA using the ReverTra Ace qPCR RT Master Mix (TOYOBO). qPCR was performed using SYBR Green and CFX96 Touch Real‐Time PCR (Bio‐Rad Laboratories, Hercules, CA). The amount of mRNA of the target gene in the sample was calculated by comparison with standard samples. Target gene expression levels were normalized to the level of *GAPDH*, as previously reported.^28^ RT‐qPCR primer sequences^29^ are listed in Table S3.

### 2.6 Western blot analysis

Western blotting was performed as previously reported^30^ using specific monoclonal antibodies against TFPI2 (#sc‐48380, Santa Cruz Biotechnology). Expression of actin (#ab14128, Abcam) was measured as a loading control. The antibodies against TFPI2 and actin were used at a 1:100 dilution and a 1:5000 dilution, respectively. Whole cells were lysed with sodium dodecyl sulfate sampling buffer. The protein signals were detected using Luminescent Image Analyzer LAS‐3000 (Fuji Film).

### 2.7 Invasion assay

Invasion assay was performed according to the recommended protocol using a Corning BioCoatTM Matrigel Invasion Chamber; 25 000 cells per well were seeded after 2 hours of gel hydration and infiltrated cells were observed after 22 hours of incubation. NIH‐ImageJ software^31^ was used for cell count.

### 2.8 Statistical analysis

Correlations between clinicopathological features and methylation subgroups were analyzed using the Fisher's exact test. Overall survival (OS) was measured from the day of the surgery to the date of MM‐related death. The Kaplan‐Meier method was used to estimate OS distribution, and differences between the groups were tested by log‐rank test. These calculations were performed using GraphPad Prism version 7.04 for Windows, GraphPad Software, www.graphpad.com. Unsupervised two‐way hierarchical clustering was performed using *R* software (www.r‐project.org/). Outcomes were recorded as statistically significant when *P* < .05.

### 2.9 Data access

Infinium 450k data on 51 clinical MM specimens and cell lines were submitted to the GEO database under the accession number GSE140171 (GSM4155681‐GSM4155734).

## 3 RESULTS

### 3.1 BRAF and NRAS mutations

**FIGURE 1:**
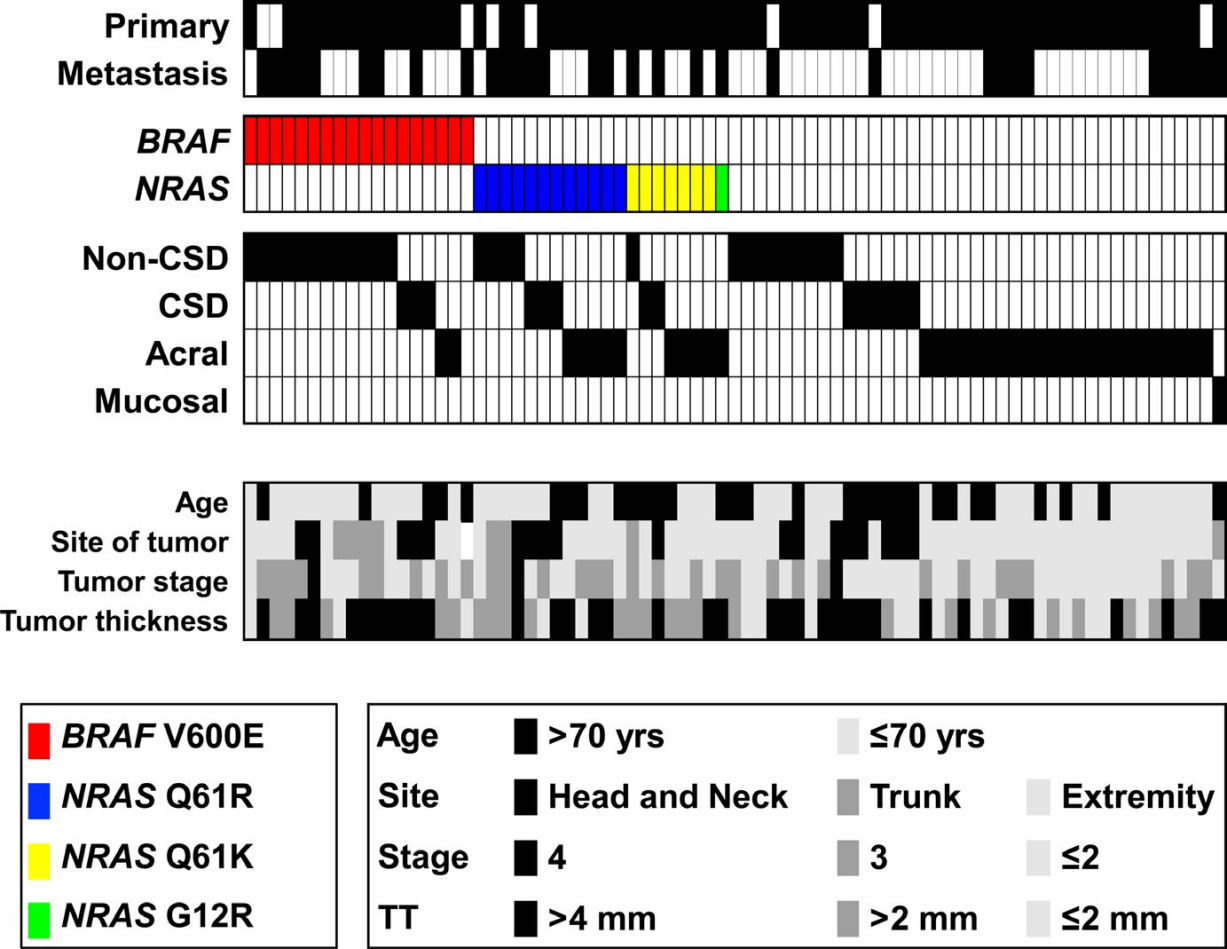
Patient and sample information. When both primary and metastatic lesions are available, the primary lesion is used for the subsequent molecular analysis. Mutations in driver genes, *BRAF* codon 600, and *NRAS* codons 12, 13, and 61 were analyzed using pyrosequencing. Among 77 cases, 35 were acral cases and less frequently observed in *BRAF*‐mut(+) MM

*BRAF* mutations were found in 18 of the 77 patients, two thirds of which occurred in non‐CSD, which is consistent with previous reports about the Japanese population^32^ (Figure 1). *NRAS* mutations were found in 20 of the 77 patients, most of which occurred at codon 61, which was more frequent than previously reported.^33^ Of the 77 cases, there were 35 acral cases, and this was considered to be an Asian patient‐specific characteristic.

### 3.2 Identification of two methylation subtypes in melanoma

**FIGURE 2:**
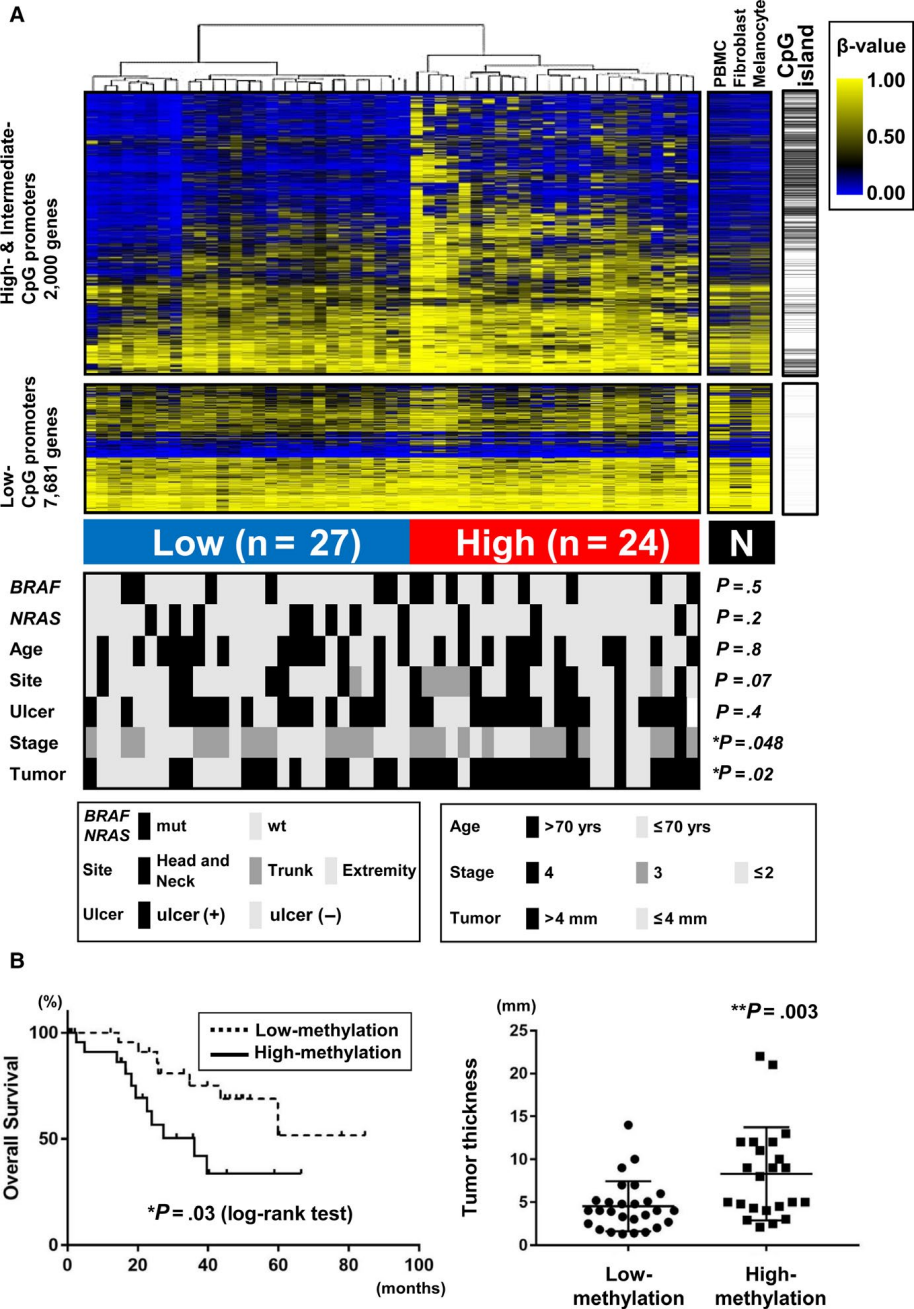
Methylation subgroups in MM. (A) Unsupervised hierarchical clustering of MM. Using Infinium 450k, 51 MM samples were subjected to methylome analysis and unsupervised clustering analysis (*top*). *PBMC*, peripheral blood mononuclear cells. The Infinium probe nearest to transcriptional start site (TSS) was selected for each gene, and used for subsequent analyses. Among the high‐ and intermediate‐CpG promoters (CpG scores >0.72 and >0.48, respectively), 2000 gene promoters with standard deviations of >0.15 in 51 samples were used for clustering analysis. 7681 genes with low‐CpG promoters (CpG score <0.48) were additionally shown. MM was stratified into two major clusters: high‐ and low‐methylation subgroups. Clinicopathological factors (*bottom*), and their association with methylation subtypes (*bottom*, *right*), were analyzed by Fisher's exact test. *Stage*, clinical stages 1‐4. *Tumor*, Breslow's tumor thickness. High‐methylation MM significantly correlated with advanced tumor progression, for example, clinical stage (*P* = .048, Fisher exact test) and Breslow's tumor thickness (*P* = .02). (B) Correlation between high‐methylation subgroup and advanced tumor progression. Tumors in high‐methylation subgroup showed significantly worse prognosis (*P* = .03, log‐rank test) (*left*) and were significantly thicker (*P* = .003, *t* test) (*right*). **P < .01, *P < .05

Comprehensive methylation analysis was performed using Infinium 450k array for 51 cases whose quality was confirmed using the FFPE QC Kit. The probe nearest to the TSS was treated as a representative probe of the gene promoter. Among the high‐ and intermediate‐CpG promoters (CpG scores >0.72 and >0.48, respectively), 2000 gene promoters with standard deviations of >0.15 in 51 samples were extracted and used for unsupervised hierarchical clustering analysis. MM was stratified into two major clusters: high‐ and low‐methylation subgroups (Figure 2A). The probes showing differentially methylated levels between the two subtypes were mostly located in CpG islands. Low‐CpG gene promoters with a CpG score <0.48 showed smaller differences in methylation status compared with high‐ and intermediate‐CpG promoters.

Correlation between methylation status and clinicopathological factors was analyzed. Although hotspot mutations of *BRAF* and *NRAS*, age, tumor site, and presence of ulcers did not show significant correlation with methylation status, high‐methylation subgroup significantly correlated with advanced stage of disease (*P* = .048, Fisher's exact test) and the presence of a thicker tumor (*P* = .02) (Figure 2A). As for the correlation of methylation status with prognosis or quantitative tumor thickness, high‐methylation subgroup significantly correlated with worse prognosis *(P* = .03, log‐rank test) and the presence of a thicker tumor (*P* = .003, *t* test) (Figure 2B).

### 3.3 Genes that define methylation subgroups

**FIGURE 3:**
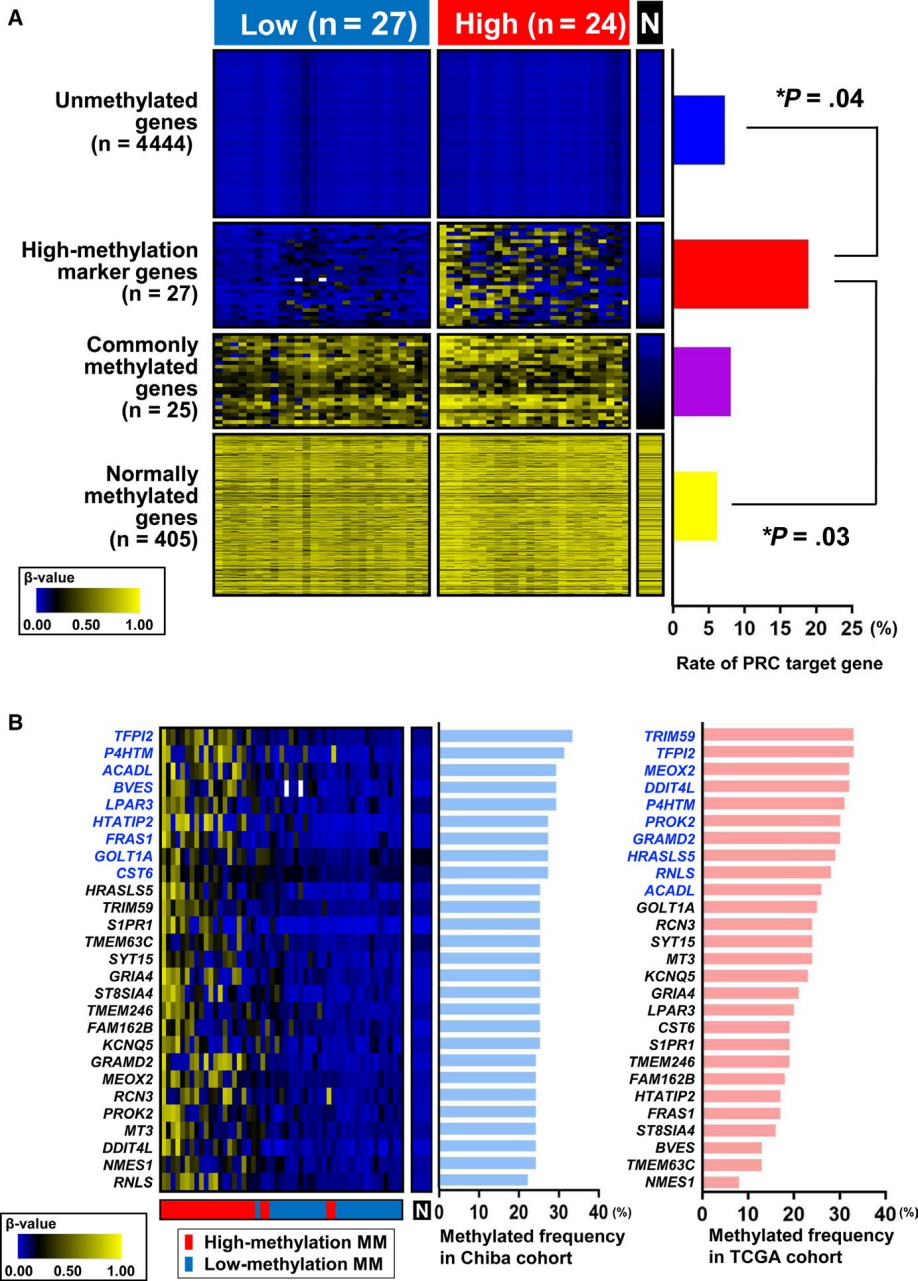
Extraction of methylation marker genes. (A) Classifier marker genes for methylation subtypes. Four categories of marker genes were extracted: (a) unmethylated genes that were unmethylated in normal samples and both high‐ and low‐methylation MM samples, (b) high‐methylation marker genes that were unmethylated in normal samples but specifically hypermethylated in high‐methylation MM, (c) commonly methylated genes that were unmethylated in normal samples but commonly hypermethylated in both high‐ and low‐methylation MM, and (d) normally methylated genes that were all methylated in normal samples and high‐ and low‐methylation MM. PRC target genes in ES cells were significantly enriched in high‐methylation marker genes, compared with unmethylated genes (*P* = .04, Fisher's exact test) and normally methylated genes (*P* = .03). *P < .05. (B) List of high‐methylation marker genes. Frequencies of aberrant hypermethylation in MM samples were shown for Chiba cohort (*pale blue*) and the TCGA cohort (*orange*). Genes hypermethylated in MM frequently at >25% are shown in *blue*

**FIGURE 4:**
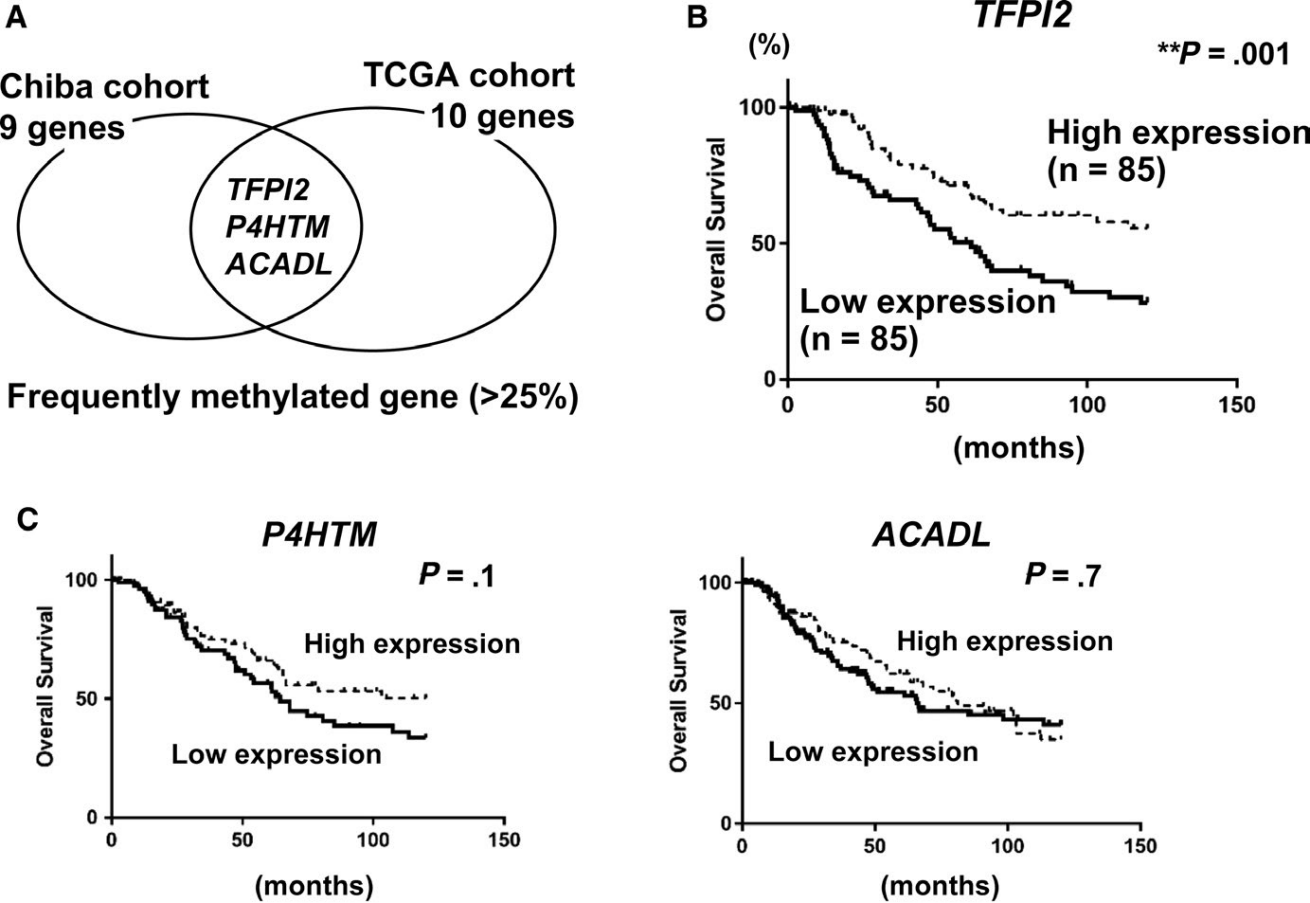
Assessment of frequently hypermethylated genes. (A) Frequently hypermethylated genes in both cohorts. *TFPI2*, *P4HTM*, and *ACADL* were found to be hypermethylated frequently (>25%) in the two cohorts commonly. (B) Significant correlation between expression of *TFPI2* and overall survival. Among 342 MM cases in the TCGA cohort for which clinical data were available, cases with top 25% expression levels of *TFPI2* were defined as the high expression group, and those with bottom 25% expression levels as the low expression group. Low expression of *TFPI2* significantly correlated with worse prognosis (*P* = .001, log‐rank test). (B) Correlation between other frequently hypermethylated genes and overall survival. High and low expression groups were defined similarly for *P4HTM* and *ACADL* using the TCGA cohort. Low expression of these genes, however, did not significantly correlate with worse prognosis

Four groups of classifier marker genes that stratify MM cases into two methylation subgroups were extracted: 4444 unmethylated genes, 27 high‐methylation marker genes, 25 commonly methylated genes, and 405 normally methylated genes (Figure 3A). Polycomb repressive complex (PRC) target genes in embryonic stem (ES) cells were significantly enriched in high‐methylation marker genes, compared with unmethylated genes (*P* = .04, Fisher's exact test) and normally methylated genes (*P* = .03) (Figure 3A). This is consistent with previous reports that genes involved in development and differentiation are PRC targets in ES cells, and that these genes are frequently hypermethylated in many types of cancers.^34^, ^35^, ^36^ The 27 high‐methylation marker genes extracted were specifically hypermethylated in high‐methylation subgroup, and their methylation frequencies in MM cases were 22%‐33% in Chiba cohort and 8%‐35% in the TCGA cohort (Figure 3B). *TFPI2*, *P4HTM*, and *ACADL* were found to be hypermethylated frequently (>25%) in both cohorts (Figure 4A).

We next analyzed the correlation between gene expression and prognosis. A significant association was observed between the worse prognosis with low expression of *TFPI2* (Figure 4B), which was the gene most frequently hypermethylated in the Chiba cohort (Figure 3B). Low expression of the other two genes, *P4HTM* and *ACADL*, did not significantly correlate with worse prognosis (Figure 4C). Therefore, we focused on *TFPI2* for the subsequent function analysis.

### 3.4 Function analysis of TFPI2

**FIGURE 5:**
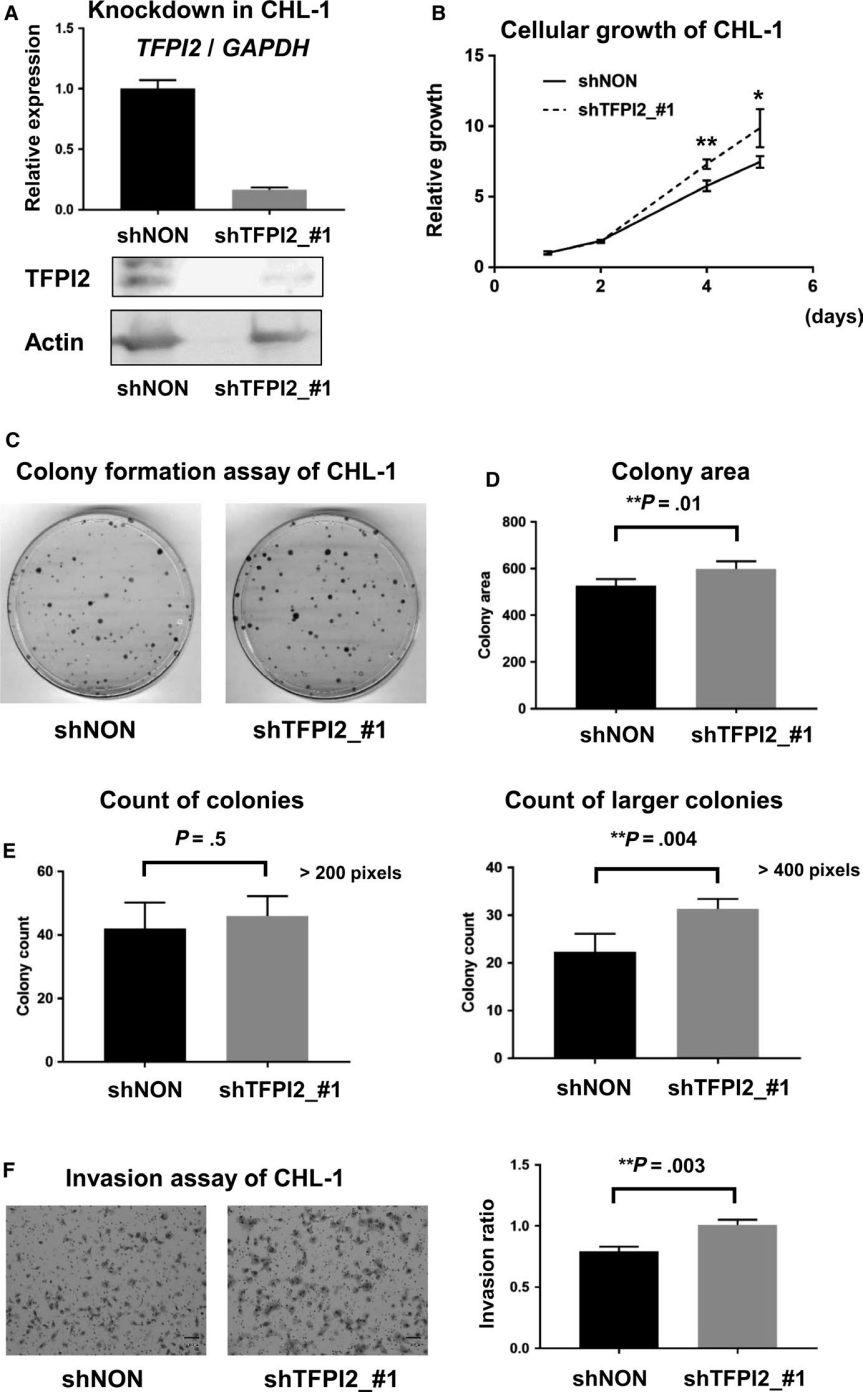
Functional analysis of *TFPI2*. (A) Knockdown of TFPI2. CHL‐1 MM cell lines were treated with shTFPI2_#1, and decreased expression of TFPI2 was confirmed by RT‐qPCR (*top*) and western blot (*bottom*), compared with shNON‐treated cells. (B) Cell proliferation assay using WST‐8. 2000 cells was seeded per well on day 0 and cultured for 5 days. Cellular growth was significantly increased in TFPI2‐knockdown CHL‐1 cells. *P < .05, **P < .01 (*t* test for triplicated experiments). (C) Representative images of colony formation assay. 500 cells were seeded per 100‐mm dish on day 0, and were fixed and stained on day 13. (D) Colony area measurement. Colonies with >200 pixels were assessed. Colony area was significantly larger in TFPI2‐knockdown CHL‐1 cells. (E) Colony count measurement. The numbers of colonies with >200 pixels and >400 pixels were counted and shown. While there was no significant difference in count of colonies with >200 pixels, TFPI2‐knockdown CHL‐1 cells formed significantly large number of colonies with >400 pixels, compared with shNON‐treated cells. (F) Invasion assay. Ratio of the number of cells that passed through a gel‐coated well to the number of cells that passed through a control well was calculated and shown. TFPI2‐knockdown CHL‐1 cells showed a significantly increased invasion ratio, compared with shNON‐treated cells. **P < .01 (*t* test for triplicated experiments)

We generated shRNA against *TFPI2* (shTFPI2_#1) to knockdown its expression in CHL‐1 MM cell line (Figure 5A). Decreased expression of *TFPI2* was confirmed at the mRNA level by RT‐qPCR, and also at the protein level by western blot, compared with shNON‐treated CHL‐1 cells. Cell proliferation was significantly increased in TFPI2‐knockdown CHL‐1 cells on days 4 (*P* = .0007) and 5 (*P* = .04) (Figure 5B).

We next conducted colony formation assays and assessed colonies with > 200 pixels (Figure 5C). The colony area was significantly larger in TFPI2‐knockdown CHL‐1 cells compared with shNON‐treated CHL‐1 cells (Figure 5D). However, there was no significant difference in the colony count when we counted the number of all colonies with >200 pixels (Figure 5E). When colonies with >400 pixels were counted, TFPI2‐knockdown CHL‐1 cells formed a significantly larger number of colonies, compared with shNON‐treated cells. These suggest that the increase of colony area is not due to the increased ability of colony formation, but due to the increase of cell proliferation.

As for the invasion assay, we calculated the ratio of the number of cells that passed through a gel‐coated well to the number of cells that passed through a control well, and thereby defined the invasion ratio. TFPI2‐knockdown CHL‐1 cells showed significantly increased invasion ratios compared with shNON‐treated cells (*P* = .03, *t* test) (Figure 5F).

**FIGURE 6:**
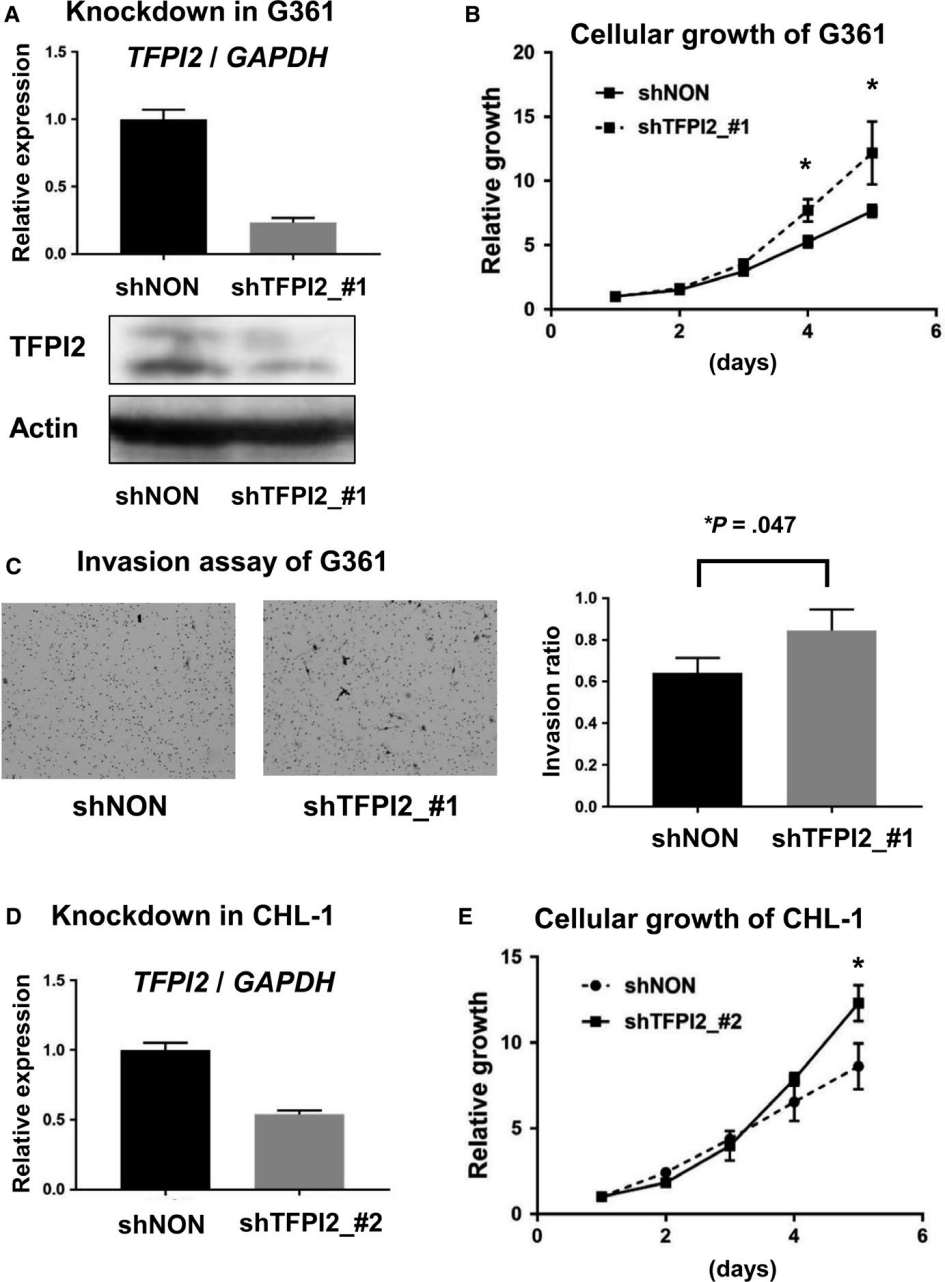
Validation analyses of TFPI2 knockdown. (A) Knockdown of TFPI2 in G361. G361 MM cell lines were treated with shTFPI2_#1, and decreased expression of TFPI2 was confirmed by RT‐qPCR (*top*) and western blot (*bottom*). (B) Cell proliferation assay using WST‐8. 2000 cells were seeded per well on day 0 and cultured for 5 days. Cellular growth was significantly increased in TFPI2‐knockdown G361 cells. *P < .05 (*t* test for triplicated experiments). (C) Invasion assay. TFPI2‐knockdown G361 cells showed a significantly increased invasion ratio, compared with shNON‐treated cells. *P < .05 (*t* test for triplicated experiments). (D) Knockdown of TFPI2 by shTFPI2_#2. Another shRNA against TFPI2 was generated to knockdown TFPI2. Decreased expression of *TFPI2* by shTFPI2_#2 was confirmed by RT‐qPCR. (E) Cell proliferation assay using WST‐8. 2000 cells were seeded per well on day 0 and cultured for 5 days. Cellular growth was significantly increased in CHL‐1 cells treated with shTFPI2_#2. *P < .05 (*t* test for triplicated experiments)

To validate these results of the increases of cell proliferation and invasion, knockdown experiments using shTFPI2_#1 were performed utilizing a different MM cell line, G361. Decreased expression of *TFPI2* was confirmed at the mRNA level by RT‐qPCR and also at the protein level by western blot, compared with shNON‐treated G361 cells (Figure 6A). Cellular growth of TFPI2‐knockdown G361 cells was confirmed to be significantly increased on days 4 (*P* = .01) and 5 (*P* = .03) (Figure 6B). Invasion ratio of TFPI2‐knockdown G361 cells was also confirmed to be significantly increased, compared with shNON‐treated cells (*P* = .047, *t* test) (Figure 6C).

We further generated another shRNA against *TFPI2* (shTFPI2_#2) for validation. Decreased expression of *TFPI2* in CHL‐1 cells was confirmed by RT‐qPCR, compared with shNON‐treated cells (Figure 6D). Cellular growth was confirmed to be significantly increased (*P* = .02) (Figure 6E).

## 4 DISCUSSION

Methylome studies in MM often compared malignant tumors with benign pigmented nevus,^37^, ^38^ and often stratified MM cases utilizing methylation information of CpG sites in the entire genome.^22^ We hereby focused on the CpG sites in the promoter region in the vicinity of TSSs and stratified patients according to the methylation status of the critical 5' regions of the genes. We stratified MM cases into two methylation subgroups and showed a significant association between the progressive phenotype of tumor thickness and overall prognosis. Among methylation marker genes extracted to define these subgroups, *TFPI2* was the most frequently hypermethylated gene in MM, low expression of which significantly correlated with poorer prognosis of MM, and knockdown of *TFPI2* led to increased cellular growth and invasion of MM.

Cancers other than MM, including colorectal cancer,^26^ gastric cancer,^39^ and oropharyngeal squamous cell carcinoma,^40^ have also been reportedly stratified into several molecular subtypes based on the methylation status of promoter regions. Methylation target genes in these malignant tumors showed significant enrichment of PRC target genes in ES cells.^36^, ^39^, ^40^ A subtype of gastric cancer with Epstein‐Barr virus infection is an exception that involves aberrant hypermethylation in non‐PRC target genes as well as PRC target genes.^39^ In the present study, we focused on methylation status at promoter regions in MM and, accordingly, stratified MM into two groups: high‐ and low‐methylation subgroups. When methylation marker genes were subsequently extracted, it was observed that high‐methylation marker genes were specifically hypermethylated in high‐methylation MM, and not in low‐methylation MM or in normal samples. Furthermore, high‐methylation marker genes showed significant enrichment of PRC target genes in ES cells, compared with unmethylated genes (unmethylated in all the samples) and normally methylated genes (methylated in all the samples). Frequent promoter hypermethylation was found to occur significantly in a subgroup of MM, and PRC target genes in ES cells were found to be preferentially hypermethylated in the high‐methylation subgroup of MM, similar to most other malignant tumors.

As for clinicopathological factors, methylation phenotypes of MM did not associate with genetic mutations, such as *BRAF* and *NRAS*, age, tumor site, or the presence of ulcers. But higher tumor stage and greater tumor thickness significantly associated with high‐methylation subgroup of MM. Tumor thickness was significantly thicker and OS was significantly shorter in high‐methylation subgroups in our cohort. These results are consistent with previous reports on MM. Thomas et al analyzed DNA methylation in 47 clinical cases and found a correlation between hypermethylation and tumor thickness.^21^ Their study used GoldenGate Cancer Panel that allowed analysis of 1402 CpG sites covering 793 cancer‐related genes, whereas our study used HumanMethylation450 BeadChip that allowed analysis of 480 000 CpG sites covering 99% of the Refseq genes. TCGA also used HumanMethylation450 BeadChip and stratified MM into four subgroups based on the analysis of CpG methylation in the entire genomic regions; a subgroup with CpG island methylation exhibited poor prognosis compared with normal‐like group.^12^ However, the result might possibly be inaccurate because the position of the probes, such as promoters, enhancers, and gene body, was not considered, and because variable number of probes were used per gene. Therefore, we conducted methylome analysis focusing on the probe nearest to the TSS of each gene with high‐ or intermediate‐CpG promoter. As for *TFPI2*, the most frequently hypermethylated marker gene, for example 21 probes were designed for the gene promoter region, and the probe nearest to TSS, at 15 bp upstream, was used for analysis. While we focused on hypermethylation of such critical CpG sites that could presumably cause silencing of downstream genes, we showed consistent results that high‐methylation MM cases correlated with progressive phenotypes of tumor, such as thicker tumors and overall shorter survival.

Methylation‐associated silencing of tumor suppressor genes, for example *COL1A2*,^41^ *RASSF1A*,^42^ *PTEN*,^19^ *KIT*,^43^ and *DDIT4L*,^44^ has been previously reported in MM. Muthusamy et al reported that methylation of *COL1A2* was observed in 80% of clinical MM cases, and its methylation was also reported in breast cancer and hepatocellular carcinoma.^41^ Koga *et al* reported that more advanced tumors had higher methylation rates for *COL1A2* and *DDIT4L*.^20^ In the present study, we showed *DDIT4L* as one of the high‐methylation marker genes that were specifically hypermethylated in high‐methylation MM subgroup exhibiting the progressive tumor phenotype.

Among high‐methylation marker genes, *TFPI2* was found to be most frequently hypermethylated in MM, and its downregulation significantly correlated with worse prognosis. Tumor‐related functions of *TFPI2* have been reported in other malignancies. While *TFPI2* is downregulated or lost during tumor progression in human glioma, induction of *TFPI2* in glioma cells resulted in less invasive phenotype.^45^ In pancreatic ductal cancer, inactivation of *TFPI2* was shown to contribute to cell proliferation, migration, and invasion.^46^ In MM, however, hypermethylation of *TFPI2* was reportedly shown in metastasis‐derived melanoma cell lines,^47^ while the tumorigenic roles of *TFPI2* in MM have not been fully investigated. In the present study, we not only demonstrated frequent hypermethylation in the vicinity of *TFPI2* TSS in clinical human MM cases, but also conducted knockdown of *TFPI2* in two MM cell lines, CHL‐1 and G361, resulting in significant increase of cellular growth and invasion. These indicate that methylation‐associated silencing of *TFPI2* plays a causal role in advanced tumor phenotypes of high‐methylation MM, which results in thicker progression, worse prognosis, and reportedly, metastasis through increasing cellular growth and invasion.

Mechanism(s) to induce aberrant DNA methylation in MM are yet to be clarified. Mutation of genes involved in epigenetic modification may be a candidate, as *IDH1/2* mutation reportedly causes high‐methylation phenotype of glioma, so‐called G‐CIMP.^48^ Furthermore, environmental factors such as exposure in early life to ambient ultraviolet (UV) may induce gene mutations.^49^ TCGA reported that *IDH1* and *ARID2* mutations were observed significantly more frequently in the subgroup of MM with high methylation of CpG islands.^12^ Further analysis is necessary to elucidate the presence of mutations of these epigenetic modifiers and its association with environmental factors, as well as in vivo assessment of methylation target genes, for example *TFPI2*.

In summary, we showed in this study that MM can be stratified into at least two subgroups based on promoter methylation status, that high‐methylation subgroup exhibits advanced tumor phenotypes, characterized by thicker progression and worse prognosis, and that methylation of *TFPI2* contributes to such progressive phenotype of MM.

## CONFLICT OF INTEREST

The authors have no conflict of interest to disclose.

## AUTHOR CONTRIBUTION

YY, HM, and AK designed the study. YY, KM, and BR performed experiments. YY and MF analyzed the data. YY prepared figures. HM and AK supervised the study. YY and AK interpreted the data and wrote the manuscript.

## Supporting information

Table S1‐S3

## Data Availability

Infinium 450k data on 51 clinical MM specimens and cell lines in GEO database under the accession number GSE140171.

## References

[1] Garbe C , Leiter U . Melanoma epidemiology and trends. Clin Dermatol. 2009;27:3‐9. 19095149 10.1016/j.clindermatol.2008.09.001

[2] Trager MH , Queen D , Samie FH , Carvajal RD , Bickers DR , Geskin LJ . Advances in prevention and surveillance of cutaneous malignancies. Am J Med. 2019;133:417‐423. 31712100 10.1016/j.amjmed.2019.10.008 PMC7709483

[3] Carr S , Smith C , Wernberg J . Epidemiology and risk factors of melanoma. Surg Clin North Am. 2020;100:1‐12. 31753105 10.1016/j.suc.2019.09.005

[4] Rastrelli M , Tropea S , Rossi CR , Alaibac M . Melanoma: epidemiology, risk factors, pathogenesis, diagnosis and classification. In Vivo. 2014;28:1005‐1011. 25398793

[5] Wang Y , Zhao Y , Ma S . Racial differences in six major subtypes of melanoma: descriptive epidemiology. BMC Cancer. 2016;16:691. 27576582 10.1186/s12885-016-2747-6 PMC5004333

[6] Fujisawa Y , Yoshikawa S , Minagawa A , et al. Clinical and histopathological characteristics and survival analysis of 4594 Japanese patients with melanoma. Cancer Med. 2019;8:2146‐2156. 30932370 10.1002/cam4.2110 PMC6536943

[7] Seynnaeve B , Lee S , Borah S , et al. Genetic and epigenetic alterations of TERT are associated with inferior outcome in adolescent and young adult patients with melanoma. Sci Rep. 2017;7:45704. 28378855 10.1038/srep45704 PMC5381111

[8] Shain AH , Bastian BC . From melanocytes to melanomas. Nat Rev Cancer. 2016;16:345‐358. 27125352 10.1038/nrc.2016.37

[9] Shain AH , Yeh I , Kovalyshyn I , et al. The genetic evolution of melanoma from precursor lesions. N Engl J Med. 2015;373:1926‐1936. 26559571 10.1056/NEJMoa1502583

[10] Davies H , Bignell GR , Cox C , et al. Mutations of the BRAF gene in human cancer. Nature. 2002;417:949‐954. 12068308 10.1038/nature00766

[11] Curtin JA , Busam K , Pinkel D , Bastian BC . Somatic activation of KIT in distinct subtypes of melanoma. J Clin Oncol. 2006;24:4340‐4346. 16908931 10.1200/JCO.2006.06.2984

[12] Akbani R , Akdemir K , Aksoy B , et al. Genomic classification of cutaneous melanoma. Cell. 2015;161:1681‐1696. 26091043 10.1016/j.cell.2015.05.044 PMC4580370

[13] Harbst K , Lauss M , Cirenajwis H , et al. Multiregion whole‐exome sequencing uncovers the genetic evolution and mutational heterogeneity of early‐stage metastatic melanoma. Cancer Res. 2016;76:4765‐4774. 27216186 10.1158/0008-5472.CAN-15-3476

[14] Hayward NK , Wilmott JS , Waddell N , et al. Whole‐genome landscapes of major melanoma subtypes. Nature. 2017;545:175‐180. 28467829 10.1038/nature22071

[15] Ting AH , McGarvey KM , Baylin SB . The cancer epigenome–components and functional correlates. Genes Dev. 2006;20:3215‐3231. 17158741 10.1101/gad.1464906

[16] Baylin SB , Jones PA . A decade of exploring the cancer epigenome ‐ biological and translational implications. Nat Rev Cancer. 2011;11:726‐734. 21941284 10.1038/nrc3130 PMC3307543

[17] Jones PA , Issa JP , Baylin S . Targeting the cancer epigenome for therapy. Nat Rev Genet. 2016;17:630‐641. 27629931 10.1038/nrg.2016.93

[18] Gonzalgo ML , Bender CM , You EH , et al. Low frequency of p16/CDKN2A methylation in sporadic melanoma: comparative approaches for methylation analysis of primary tumors. Cancer Res. 1997;57:5336‐5347. 9393758

[19] Mirmohammadsadegh A , Marini A , Nambiar S , et al. Epigenetic silencing of the PTEN gene in melanoma. Cancer Res. 2006;66:6546‐6552. 16818626 10.1158/0008-5472.CAN-06-0384

[20] Koga Y , Pelizzola M , Cheng E , et al. Genome‐wide screen of promoter methylation identifies novel markers in melanoma. Genome Res. 2009;19:1462‐1470. 19491193 10.1101/gr.091447.109 PMC2720187

[21] Thomas NE , Slater NA , Edmiston SN , et al. DNA methylation profiles in primary cutaneous melanomas are associated with clinically significant pathologic features. Pigment Cell Melanoma Res. 2014;27:1097‐1105. 24986547 10.1111/pcmr.12289 PMC4211983

[22] Lauss M , Ringnér M , Karlsson A , et al. DNA methylation subgroups in melanoma are associated with proliferative and immunological processes. BMC Med Genomics. 2015;8:73. 26545983 10.1186/s12920-015-0147-4 PMC4636848

[23] Seymour MT , Brown SR , Middleton G , et al. Panitumumab and irinotecan versus irinotecan alone for patients with KRAS wild‐type, fluorouracil‐resistant advanced colorectal cancer (PICCOLO): a prospectively stratified randomised trial. Lancet Oncol. 2013;14:749‐759. 23725851 10.1016/S1470-2045(13)70163-3 PMC3699713

[24] Takane K , Fukuyo M , Matsusaka K , et al. The frequency of promoter DNA hypermethylation is decreased in colorectal neoplasms of familial adenomatous polyposis. Oncotarget. 2018;9:32653‐32666. 30220972 10.18632/oncotarget.25987 PMC6135695

[25] Weber M , Hellmann I , Stadler MB , et al. Distribution, silencing potential and evolutionary impact of promoter DNA methylation in the human genome. Nat Genet. 2007;39:457‐466. 17334365 10.1038/ng1990

[26] Yagi K , Akagi K , Hayashi H , et al. Three DNA methylation epigenotypes in human colorectal cancer. Clin Cancer Res. 2010;16:21‐33. 20028768 10.1158/1078-0432.CCR-09-2006

[27] Hata A , Nakajima T , Matsusaka K , et al. A low DNA methylation epigenotype in lung squamous cell carcinoma and its association with idiopathic pulmonary fibrosis and poorer prognosis. Int J Cancer. 2020;146:388‐399. 31241180 10.1002/ijc.32532

[28] Kaneda A , Fujita T , Anai M , et al. Activation of Bmp2‐Smad1 signal and its regulation by coordinated alteration of H3K27 trimethylation in Ras‐induced senescence. PLoS Genet. 2011;7:e1002359. 22072987 10.1371/journal.pgen.1002359 PMC3207904

[29] Mino K , Nishimura S , Ninomiya S , et al. Regulation of tissue factor pathway inhibitor‐2 (TFPI‐2) expression by lysine‐specific demethylase 1 and 2 (LSD1 and LSD2). Biosci Biotechnol Biochem. 2014;78:1010‐1017. 25036127 10.1080/09168451.2014.910104

[30] Kita K , Sugita K , Sato C , Sugaya S , Sato T , Kaneda A . Extracellular release of Annexin A2 is enhanced upon oxidative stress response via the p38 MAPK pathway after low‐dose X‐ray irradiation. Radiat Res. 2016;186:79‐91. 27356027 10.1667/RR14277.1

[31] Schneider CA , Rasband WS , Eliceiri KW . NIH Image to ImageJ: 25 years of image analysis. Nat Methods. 2012;9:671‐675. 22930834 10.1038/nmeth.2089 PMC5554542

[32] Sakaizawa K , Ashida A , Uchiyama A , et al. Clinical characteristics associated with BRAF, NRAS and KIT mutations in Japanese melanoma patients. J Dermatol Sci. 2015;80:33‐37. 26282084 10.1016/j.jdermsci.2015.07.012

[33] Uhara H , Ashida A , Koga H , et al. NRAS mutations in primary and metastatic melanomas of Japanese patients. Int J Clin Oncol. 2014;19:544‐548. 23739925 10.1007/s10147-013-0573-2

[34] Easwaran H , Johnstone SE , Van Neste L , et al. A DNA hypermethylation module for the stem/progenitor cell signature of cancer. Genome Res. 2012;22:837‐849. 22391556 10.1101/gr.131169.111 PMC3337430

[35] Ku M , Koche RP , Rheinbay E , et al. Genomewide analysis of PRC1 and PRC2 occupancy identifies two classes of bivalent domains. PLoS Genet. 2008;4:e1000242. 18974828 10.1371/journal.pgen.1000242 PMC2567431

[36] Widschwendter M , Fiegl H , Egle D , et al. Epigenetic stem cell signature in cancer. Nat Genet. 2007;39:157‐158. 17200673 10.1038/ng1941

[37] Gao L , van den Hurk K , Moerkerk PTM , et al. Promoter CpG island hypermethylation in dysplastic nevus and melanoma: CLDN11 as an epigenetic biomarker for malignancy. J Invest Dermatol. 2014;134:2957‐2966. 24999589 10.1038/jid.2014.270

[38] Micevic G , Theodosakis N , Bosenberg M . Aberrant DNA methylation in melanoma: biomarker and therapeutic opportunities. Clin Epigenetics. 2017;9:34. 28396701 10.1186/s13148-017-0332-8 PMC5381063

[39] Matsusaka K , Kaneda A , Nagae G , et al. Classification of Epstein‐Barr virus‐positive gastric cancers by definition of DNA methylation epigenotypes. Cancer Res. 2011;71:7187‐7197. 21990320 10.1158/0008-5472.CAN-11-1349

[40] Nakagawa T , Matsusaka K , Misawa K , et al. Frequent promoter hypermethylation associated with human papillomavirus infection in pharyngeal cancer. Cancer Lett. 2017;407:21‐31. 28823962 10.1016/j.canlet.2017.08.008

[41] Muthusamy V , Duraisamy S , Bradbury CM , et al. Epigenetic silencing of novel tumor suppressors in malignant melanoma. Cancer Res. 2006;66:11187‐11193. 17145863 10.1158/0008-5472.CAN-06-1274

[42] Tellez CS , Shen L , Estécio MRH , Jelinek J , Gershenwald JE , Issa J‐PJ . CpG island methylation profiling in human melanoma cell lines. Melanoma Res. 2009;19:146‐155. 19441164 10.1097/cmr.0b013e32832b274e

[43] Dahl C , Abildgaard C , Riber‐Hansen R , Steiniche T , Lade‐Keller J , Guldberg P . KIT is a frequent target for epigenetic silencing in cutaneous melanoma. J Invest Dermatol. 2015;135:516‐524. 25178104 10.1038/jid.2014.372

[44] Furuta J , Nobeyama Y , Umebayashi Y , Otsuka F , Kikuchi K , Ushijima T . Silencing of Peroxiredoxin 2 and aberrant methylation of 33 CpG islands in putative promoter regions in human malignant melanomas. Cancer Res. 2006;66:6080‐6086. 16778180 10.1158/0008-5472.CAN-06-0157

[45] Konduri SD , Rao CN , Chandrasekar N , et al. A novel function of tissue factor pathway inhibitor‐2 (TFPI‐2) in human glioma invasion. Oncogene. 2001;20:6938‐6945. 11687973 10.1038/sj.onc.1204847

[46] Sato N , Parker AR , Fukushima N , et al. Epigenetic inactivation of TFPI‐2 as a common mechanism associated with growth and invasion of pancreatic ductal adenocarcinoma. Oncogene. 2005;24:850‐858. 15592528 10.1038/sj.onc.1208050

[47] Nobeyama Y , Okochi‐Takada E , Furuta J , et al. Silencing of tissue factor pathway inhibitor‐2 gene in malignant melanomas. Int J Cancer. 2007;121:301‐307. 17372906 10.1002/ijc.22637

[48] Turcan S , Rohle D , Goenka A , et al. IDH1 mutation is sufficient to establish the glioma hypermethylator phenotype. Nature. 2012;483:479‐483. 22343889 10.1038/nature10866 PMC3351699

[49] Garibyan L , Fisher DE . How sunlight causes melanoma. Curr Oncol Rep. 2010;12:319‐326. 20623386 10.1007/s11912-010-0119-y

