# Supplementary material for: Higher methylation subtype of malignant melanoma and its correlation with thicker progression and worse prognosis
Source: Cancer Med. 2020 May 14;9(19):7194–204. doi: 10.1002/cam4.3127 (PMC7541157; doi:10.1002/cam4.3127)
Supplement: Supplementary file 1 — Table S1‐S3 [file CAM4-9-7194-s001.docx]

**Supporting Information**

**Higher methylation subtype of malignant melanoma and its correlation with thicker progression and worse prognosis**

**Supporting Tables:**

**Supporting Table S1**

**Supporting Table S2**

**Supporting Table S3**

**Supporting Table S1.** Primers for *BRAF* and *NRAS* hotspot mutation analysis.

| Region of interest | Primer types | Primer Sequence |
| --- | --- | --- |
| *BRAF* codon 600 | Forward | TGAAGACCTCACAGTAAAAATAGG |
|  | Reverse* | TCCAGACAACTGTTCAAACTGAT |
|  | Sequencing | TGATTTTGGTCTAGCTACA |
| *NRAS* codons 12 and 13 | Forward | CTTGCTGGTGTGAAATGACTGAG |
|  | Reverse* | TGGATTGTCAGTGCGCTTTTC |
|  | Sequencing | CTGGTGGTGGTTGGA |
| *NRAS* codon 61 | Forward* | GAAACCTGTTTGTTGGACATACTG |
|  | Reverse | TCGCCTGTCCTCATGTATTG |
|  | Sequencing | CTCTCATGGCACTGTACT |

* Primers with 5ʹ-biotin tag.

**Supporting Table S2.** Sequences of oligo DNA for knockdown.

| shRNA | Strand | Sequence |
| --- | --- | --- |
| shTFPI2_#1 | top | CCGGGCCTTTATGGTTGTATCTGAACTCGAGTTCAGATACAACCATAAAGGCTTTTTG |
|  | bottom | AATTCAAAAAGCCTTTATGGTTGTATCTGAACTCGAGTTCAGATACAACCATAAAGGC |
| shTFPI2_#2 | top | CCGGCCTGTGATGCTTTCACCTATACTCGAGTATAGGTGAAAGCATCACAGGTTTTTG |
|  | bottom | AATTCAAAAACCTGTGATGCTTTCACCTATACTCGAGTATAGGTGAAAGCATCACAGG |
| shNON | top | CCGGCAACAAGATGAAGAGCACCAACTCGAGTTGGTGCTCTTCATCTTGTTGTTTTTG |
|  | bottom | AATTCAAAAACAACAAGATGAAGAGCACCAACTCGAGTTGGTGCTCTTCATCTTGTTG |

Oligonucleotide sequences of shRNA against *TFPI2* (shTFPI2_#1 and _#2) and control non-target shRNA (shNON), inserted into pLKO.1 vector between EcoRI and AgeI sites, are shown, and 21-mer target sequences are underlined.

**Supporting Table S3.** Primers for RT-PCR.

| Gene | Primer types | Primer Sequence | Anneal (°C) |
| --- | --- | --- | --- |
| *TFPI2* | Forward | GTCGATTCTGCTGCTTTTCC | 62.5 |
|  | Reverse | CAGCTCTGCGTGTACCTGTC |  |
| *GAPDH* | Forward | CCAGGTGGTCTCCTCTGACTTC | 62.5 |
|  | Reverse | TCATACCAGGAAATGAGCTTGACA |  |
